# Estimation of depression, anxiety and serum cortisol in patients with oral lichen planus, leukoplakia and oral submucous fibrosis

**DOI:** 10.6026/973206300200655

**Published:** 2024-06-30

**Authors:** Nagaraja A, Yusra Khan, Dheeraj Sharma, Neeraj Sharma, Ranjeeta Mehta, Sameer Gupta

**Affiliations:** 1 Department of Oral & Maxillofacial Pathology and Oral Microbiology, Seema Dental College & Hospital, Rishikesh, Uttarakhand, India; 2 Department of Oral & Maxillofacial Pathology and Oral Microbiology, Faculty of Dentistry, Jamia Millia Islamia University, New Delhi, India; 3 Department of Oral & Maxillofacial Pathology and Oral Microbiology, Index Institute of Dental Sciences, Indore, M.P., India; 4 Departmentof Prosthodontics, Modern Dental College & Research centre, Indore, M.P., Indore, India; 5 DepartmentofOral Medicine ∓ Maxillofacial Radiology, Seema Dental College & Hospital, Rishikesh, Uttarakhand, India; 6 Department of Oral & Maxillofacial Surgery, Inderprastha Dental College and Hospital, Ghaziabad, Uttar Pradesh, India

**Keywords:** anxiety, depression, cortisol, Oral leukoplakia, oral lichen planus, OSMF

## Abstract

Stress and anxiety may be found in patients with oral submucous fibrosis (OSMF), oral leukoplakia (OL) and oral lichen planus (OLP). Cortisol, sometimes referred to as the "stress hormone," has been employed as a stress predictor. Therefore, it is of interest to estimate the levels of depression, anxiety and serum cortisol and establish correlation between them in patients with OL. OLP and OSMF. There were 240 patients, aged 20 years to 45 years, who were divided into four categories (OL, OSMF, OLP and control) of 60 patients apiece. In the supervision of a psychiatrist, the Hamilton Depression Rating Scale (HAM D) and Hamilton Anxiety Rating Scale (HAM (A) questionnaires were filled out. Five millilitres of venous blood were extracted using standard aseptic technique, and all of the samples were examined for serum cortisol level. Anxiety and depression was found in subjects of OL, OSMF and OLP at advanced stages. It was inferred that serum cortisol level was statistically correlated with depression and anxiety in patients with OL, OSMF and OLP.

## Background:

Stress is defined as a state of physical or emotional strain that might show up as physical symptoms, psychological symptoms, or both [1, 2-3]. Examples of these symptoms include fatigue, anxiousness, insomnia, and depression. Depression is regarded as "a state of dissatisfaction or sorrow," which is felt occasionally, whereas anxiety can be described as "an emotional condition that includes feeling uneasy, discomfort, and apprehension about any known or unknown threat"[4, 5- 6]. Stress weakens our resistance to infection through two different processes, which aids in the progression of disease [7, 8-9]. The primary one is a biological process that involves the synthesis of cortisol regulated by the "hypothalamic-pituitary-adrenal (HPA) axis." The other type is the cognitive mechanism that encourages harmful habits like smoking, drinking, eating poorly, not brushing your teeth, and engaging in other parafunctional activities [10, 11- 12]. As a consequence of these behaviors, there is a decline in oral health, which leads to a range of oral disorders [13, 14-15]. People acquire habits over time, such as smoking, consuming gutka, tobacco chewing, betel nut chewing, and pan chewing, which might result in the occurrence of potentially malignant illnesses (PMDs). Among the most prevalent oral mucosal disorders in humans are oral submucous fibrosis (OSMF), oral leukoplakia (OL) and oral lichen planus (OLP)[16, 17-18]. These conditions should be further studied as psychological disorders. According to some reports, as many as forty percent of cancer patients experience a considerable degree of misery [19, 20-21]. Cortisol, sometimes referred to as the "stress hormone," has been employed as a stress predictor. The primary glucocorticoid in humans, cortisol affects vascular response, metabolic processes, immunoregulation, mental processing, and personality [22, 23-24]. The significant link between psychiatric problems and chronic physical ailments have been the subject of much research in recent years. Nonetheless, there is still a dearth of information on psychological morbidity in OL, OLP and OSMF [13-18]. Therefore, it is of interest to estimate the levels of depression, anxiety and serum cortisol and establish correlation between them in patients with OL. OLP and OSMF.

## Methods and materials:

Patients who visited in the institution outpatient facility were included in the study. There were 240 patients, aged 20 years to 45 years, who were divided into four categories of 60 patients apiece (Table 1). Patients who met the following requirements were accepted into the study: they had to be ready to participate, be older than eighteen, and have an established record of smoking, using smokeless tobacco, chewing areca nut products, and smoking. PMDs of OMF, OL, and OLP that have been clinically as well as histopathological confirmed. Patients who were hesitant to participate in the trial or who were receiving current therapy for one of the disorders considered in the study were excluded from the study. Individuals with physiological situations like gestation or systemic illnesses as well as patients with impaired health, particularly those with mental health disorders were excluded. Individuals with periodontal illnesses or oral mucosal lesions were also excluded. The patients underwent routine examination procedures. In the supervision of a psychiatrist, the Hamilton Depression Rating Scale (HAM D) and Hamilton Anxiety Rating Scale (HAM (A)) questionnaires were filled out [17]. An early blood test to measure cortisol levels was planned for each patient. Five millilitres of venous blood were extracted using standard aseptic technique, and all of the samples were examined right away. Using the ROCHE COBA E 411 electrochemiluminescence immunoassay, the serum cortisol level was determined. Serum cortisol levels in the range of 138 to 600 nmol/L were considered normal.

## Statistical analysis:

Excel was used to tabulate the gathered data. Version 25.0 of the Statistical Package for Social Sciences (SPSS) for Windows was used to analyze the data (IBM Corp, Armonk, NY). There was usage of descriptive statistics like mean, standard deviation, and percentage. All parameter distributions were examined for normality using the Shapiro-Wilk test. Using the independent samples t test, variables with normal distributions in two groups were compared. One way analysis of variance with post hoc was used to compare the means of more than two groups. When data adhere to the premise of homogeneity of variances, Tukey's HSD is used; when data do not, the post hoc Games-Howell test is used. The Fisher's Freeman-Halton or Chi square tests compared frequencies, by cross tabulation precisely. The degree and direction of the relationship between anxiety and depression and blood cortisol levels were evaluated using Spearman's rank correlation. It was deemed statistically significant when P < 0.05.

## Results:

The serum cortisol level increased as the anxiety level raised from normal too mild to moderate. The serum cortisol level was statistically correlated with the level of anxiety in all study participants (Table 2).

No study participants in the category of OSMF, OL and OLP were found to have serum cortisol levels indicative of normal anxiety levels. It was observed that 45 OLP patients, 31 OL patients and 36 OSMF patients were considered to have serum cortisol level indicative of mild anxiety level. Similarly, the serum cortisol levels indicative of moderate anxiety was observed in 21 OSMF, 15 OL and 23 OLP patients. Maximum number of patients with mild anxiety level and moderate anxiety level was found in OLP category followed by OSMF category and OL category. The findings were significantly correlated (Table 3).

The mean anxiety scores were 18.42±4.42, 17.84±4.49, 19.50±4.80 and 7.71±4.67 in study participants of OSMF, OL, OLP and control category. The anxiety score was maximum in OLP patients followed by OSMF and OL (Table 4). The findings were significant statistically.

The serum cortisol level increased as the depression level raised from normal, mild, moderate, severe and very severe. The serum cortisol level was statistically correlated with depression levels (Table 5).

It was also observed that number of study participants with serum cortisol levels corresponding to mild, moderate, severe and very severe depression levels was maximum in patients with OL followed by OLP and OSMF. The findings were significant statistically (Table 6).

The mean depression score in study subjects of OSMF, OL,OLP and control was 12.47±5.34, 13.28±4.83, 12.58±6.45 and 4.11±2.27 respectively (Table 7). The depression score was maximum in patients with OL followed by OLP and OSMF. The findings were significant statistically. Anxiety and depression was found in subjects of OL, OSMF and OLP at advanced stages. It was inferred that serum cortisol level was statistically correlated with depression and anxiety in patients with OL, OSMF and OLP.

## Discussion:

Some studies have evaluated cortisol, also known as the "stress hormone," to anticipate stress. Cortisol, the main glucocorticoid in humans, influences immunoregulation, mental processing, vascular response, metabolism, and personality [12- 18].Recent years have seen a substantial amount of study on the major relationship between persistent physical diseases and psychiatric issues. However, data regarding psychological morbidity in OL, OLP, and OSMF is still scarce [14-21]. Anxiety and depression was found in all subjects of OL, OSMF and OLP. It was inferred that serum cortisol level was statistically correlated with depression and anxiety in patients with OL, OSMF and OLP. The serum cortisol level increased as the anxiety level raised from normal too mild to moderate. The serum cortisol level increased as the depression level raised from normal, mild, moderate, severe and very severe. The findings of our study are in accordance with findings of other studies showing presence of anxiety and depression in patients with OSMF, OLP and OL [15- 22]. Morever, some studies also showed increased cortisol levels in patients with greater level of anxiety and depression [17-25]. This finding is also observed in our study. Stress is characterized as a condition of emotional or physical strain that may manifest as psychological, bodily, or both symptoms. These symptoms can include despair, anxiety, sleeplessness, and weariness [16- 23]. In contrast to anxiety, which is defined as an emotional condition that includes feeling uneasy, discomfort, and apprehension about any known or unknown threat, depression is considered to be an infrequent state of dissatisfaction or sorrow [17-24]. Through two distinct mechanisms, stress reduces our ability to fight against infection, which promotes the spread of illness [11- 18].The first is a physiological mechanism involving the production of cortisol under the control of the hypothalamic-pituitary-adrenal (HPA) axis [4-8]. The other kind is the cognitive system that promotes unhealthy behaviors like binge drinking, smoking, eating poorly, skipping teeth cleanings, and other paranormal pursuits [3-7].

These habits cause the mouth health to deteriorate, which in turn causes a variety of dental illnesses[11- 16].Habits that lead to the development of potentially malignant diseases (PMDs) include smoking, ingesting gutka, chewing tobacco, chewing betel nuts, and pan chewing. These habits are acquired over time by people[12- 18].Oral lichen planus (OLP), oral leukoplakia (OL), and oral submucous fibrosis (OSMF) are three of the most common oral mucosal illnesses in humans[3-9]. Further research on these illnesses as psychiatric disorders is warranted. It has been reported that up to forty percent of cancer patients suffer to a significant extent [7-13].In our study, the mean anxiety scores were 18.42±4.42, 17.84±4.49, 19.50±4.80 and 7.71±4.67 in study participants of OSMF, OL, OLP and control category. The anxiety score was maximum in OLP patients followed by OSMF and OL. The findings were significant statistically. The mean depression score in study subjects of OSMF, OL, OLP and control was 12.47±5.34, 13.28±4.83, 12.58±6.45 and 4.11±2.27 respectively. The depression score was maximum in patients with OL followed by OLP and OSMF. The findings were significant statistically. The findings of our study is in accordance with findings of some studies showing maximum anxiety in OLP and minimum anxiety in OL [12-17]. Some studies also showed that anxiety was greater in OSMF patients as compared to OL patients. There are some studies which don't agree with findings of our study because they showed that depression was maximum in OSMF patients [11- 15]. However our study showed that depression was greater in OL patients. Current lifestyle patterns show addictive behaviors toward harmful drugs including smoking, consuming gutkha, and using nicotine. These behaviors may become more frequent when stress levels rise concurrently, which also raises the prevalence of PMDs [10- 17]. Therefore, it can be argued with some degree of confidence that psychiatric morbidity evaluation would represent the mental state of the patient, and predicted blood cortisol levels would represent the stress pattern associated with these disorders [18-24]. These two characteristics may be useful in the early detection and potential prevention of a fatal illness like cancer. Therefore, in addition to the diagnosis of these conditions, mandatory psychological counselling needs to be included in the treatment plan [12- 19]. In individuals with OSMF and leukoplakia, there was a highly significant and strong positive association observed between the levels of anxiety, depression, and serum cortisol. There is evidence to support the hypothesis that elevated depression is correlated with elevated blood cortisol levels [3-9]. The heightened levels of anxiety and sadness may have multiple causes and be linked to symptoms of open mouth syndrome (OSMF), including speech difficulties, limited mouth opening, and difficulty eating or chewing different foods[11- 18]. This needs to be supported by more focused and thorough mental assessments. Another possibility is that depression stimulates the hypothalamic pituitary adrenal axis, which raises cortisol levels in the blood by producing more corticotrophin-releasing hormone [19-25]. Any revelation of information about the existence of a PMD may cause anxiety and despair in and of it, and this has to be confirmed by additional research [12-19]. According to reports, patients with late illness stages had higher rates of psychiatric morbidity, and most OSMF patients had up to moderate levels of depression. This is comparable to the current study, which discovered a high correlation between later stages of OL, OLP and OSMF and psychological illness [20-25].

## Conclusion:

Anxiety and depression was found significantly in subjects of OL, OSMF and OLP. It was also inferred that serum cortisol level was statistically correlated with depression and anxiety in patients with OL, OSMF and OLP.

## Figures and Tables

**Table 1:** Distribution of study participants

**Category**	**Study participants**	**Number**
Category 1	Histopathological and clinically confirmed cases of OSMF	60
Category 2	Histopathological and clinically confirmed cases of OL	60
Category 3	Histopathological and clinically confirmed cases of OLP	60
Category 4	Sex and age coincided, healthy controls free of any indications or symptoms of the conditions listed above	60

**Table 2:** Data regarding of level of serum cortisol in different level of anxiety

	**Anxiety levels**		
	**Normal**	**Mild**	**Moderate**
Number	56	124	60
Mean serum cortisol level (nmol/L), Mean±SD	168.84 ±41.66	361.29±95.13	479.66±87.92
P value		0.001	

**Table 3:** Data regarding levels of anxiety study participants of different categories

	**OSMF**	**OL**	**OLP**	**Control**
Normal (n=56)	0	0	0	56
Mild (n=124)	36	31	45	12
Moderate (n=60)	21	15	23	0
P value		0.001		

**Table 4:** Mean anxiety score recorded among study participants in different categories

	**OSMF**	**OL**	**OLP**	**Control**
Mean anxiety score (Mean±SD)	18.42±4.42	17.84±4.49	19.50±4.80	7.71±4.67
P value		0.001		

**Table 5:** Data regarding of level of serum cortisol in different level of depression

	**Depression levels**				
	**Normal**	**Mild**	**Moderate**	**Severe**	**Very severe**
Number	89	71	67	7	6
Mean serum cortisol level (nmol/L), Mean±SD	227.08±84.72	363.55±75.32	463.16±93.78	513.18±123.12	663.06±40.0
P value			0.001		

**Table 6:** Data regarding levels of depression in study participants of different categories

	**OSMF**	**OL**	**OLP**	**Control**
Normal (n=89)	7	8	7	67
Mild (n=71)	14	31	21	5
Moderate (n=67)	22	24	23	0
Severe (n=7)	2	3	2	
Very severe (n=6)	2	2	2	

**Table 7:** Mean depression score recorded among study participants in different categories

	**OSMF**	**OL**	**OLP**	**Control**
Mean depression score (Mean±SD)	12.47±5.34	13.28±4.83	12.58±6.45	4.11±2.27
P value		0.001		
